# Improving Prediction of Survival for Extremely Premature Infants Born at 23 to 29 Weeks Gestational Age in the Neonatal Intensive Care Unit: Development and Evaluation of Machine Learning Models

**DOI:** 10.2196/42271

**Published:** 2024-02-14

**Authors:** Angie Li, Sarah Mullin, Peter L Elkin

**Affiliations:** 1 Department of Biomedical Informatics Jacobs School of Medicine and Biomedical Sciences University at Buffalo Buffalo, NY United States

**Keywords:** reproductive informatics, pregnancy complications, premature birth, neonatal mortality, machine learning, clinical decision support, preterm, pediatrics, intensive care unit outcome, health care outcome, survival prediction, maternal health, decision tree model, socioeconomic

## Abstract

**Background:**

Infants born at extremely preterm gestational ages are typically admitted to the neonatal intensive care unit (NICU) after initial resuscitation. The subsequent hospital course can be highly variable, and despite counseling aided by available risk calculators, there are significant challenges with shared decision-making regarding life support and transition to end-of-life care. Improving predictive models can help providers and families navigate these unique challenges.

**Objective:**

Machine learning methods have previously demonstrated added predictive value for determining intensive care unit outcomes, and their use allows consideration of a greater number of factors that potentially influence newborn outcomes, such as maternal characteristics. Machine learning–based models were analyzed for their ability to predict the survival of extremely preterm neonates at initial admission.

**Methods:**

Maternal and newborn information was extracted from the health records of infants born between 23 and 29 weeks of gestation in the Medical Information Mart for Intensive Care III (MIMIC-III) critical care database. Applicable machine learning models predicting survival during the initial NICU admission were developed and compared. The same type of model was also examined using only features that would be available prepartum for the purpose of survival prediction prior to an anticipated preterm birth. Features most correlated with the predicted outcome were determined when possible for each model.

**Results:**

Of included patients, 37 of 459 (8.1%) expired. The resulting random forest model showed higher predictive performance than the frequently used Score for Neonatal Acute Physiology With Perinatal Extension II (SNAPPE-II) NICU model when considering extremely preterm infants of very low birth weight. Several other machine learning models were found to have good performance but did not show a statistically significant difference from previously available models in this study. Feature importance varied by model, and those of greater importance included gestational age; birth weight; initial oxygenation level; elements of the APGAR (appearance, pulse, grimace, activity, and respiration) score; and amount of blood pressure support. Important prepartum features also included maternal age, steroid administration, and the presence of pregnancy complications.

**Conclusions:**

Machine learning methods have the potential to provide robust prediction of survival in the context of extremely preterm births and allow for consideration of additional factors such as maternal clinical and socioeconomic information. Evaluation of larger, more diverse data sets may provide additional clarity on comparative performance.

## Introduction

Preterm birth has long been a leading cause of infant mortality, with the lowest gestational age births associated with the highest rates of mortality [1]. In 2019, 59,506 infants were born at 31 weeks or less in the United States, and the infant mortality rate in this cohort was 18% [2]. When a patient is expected to deliver an extremely preterm infant, counseling on possible outcomes, methods of resuscitation, and anticipated course in the neonatal intensive care unit (NICU) ideally begins prior to birth. Many providers have used the National Institute of Child Health and Human Development (NICHD) risk calculator to initiate this discussion on the chances of infant mortality and severe morbidity after birth. The calculator is based on a logistic regression model using 5 prepartum factors (gestational age, estimated weight, sex, antenatal steroids, and multiple birth), derived from the preterm birth data of a network of US hospitals. With advances in NICU care and more knowledge about long-term outcomes, the calculator was updated in 2020 and maintains a similar performance (mean 0.744, SD 0.005) [3,4]. After initial resuscitation, several scoring systems are also available to predict mortality after a neonate arrives in the NICU [5-7]. However, they are less predictive with extremely low birth weight infants, as evidenced by the Score for Neonatal Acute Physiology With Perinatal Extension II (SNAPPE-II) survival model having a mean performance of 0.78 (SD 0.01) for infants weighing less than 1500 g at birth versus 0.91 (SD 0.01) overall. On review of several models, Clinical Risk Index for Babies (CRIB) had the highest performance in predicting very low birth weight neonate survival, with a mean of 0.88 (SD 0.02), although the CRIB and SNAPPE models were developed with data from geographically separate populations (Europe vs North America) [8].

Despite counseling supported by available risk calculators, decisions surrounding the continuation of life support and redirection to end-of-life care remain extremely difficult in the context of birth at the periviable preterm gestational ages because the postnatal course can be highly variable [9-11]. In addition, perceptions regarding the clinical situation can differ among providers and family members, and consideration of clinical and social context may be helpful [12,13].

Numerous machine learning models have been tested to improve the prediction of adult intensive care unit outcomes. The Medical Information Mart for Intensive Care III (MIMIC-III) database, which contains electronic health record (EHR) information of critical care patients at the Beth Israel Deaconess Medical Center from 2001 to 2012, has often been a source of data used in their development and testing [14-17]. Using the NICU data from MIMIC-III, this study builds and compares different types of machine learning algorithms that predict neonatal mortality and examines the value of incorporating features representing both structured and unstructured clinical elements for extremely preterm infants.

## Methods

### Ethical Considerations

The institutional review board of the University at Buffalo determined the study (ID STUDY00003721) to be exempt as a secondary analysis of a publicly available data set. A data use agreement was obtained for the MIMIC-III database, which contains deidentified protected health information freely available for secondary analysis. The primary data collection for MIMIC-III was originally approved by the institutional review boards of Beth Israel Deaconess Medical Center and Massachusetts Institute of Technology with a waiver of individual patient consent, and no compensation was provided at that time.

### Data Selection

**Figure 1:**
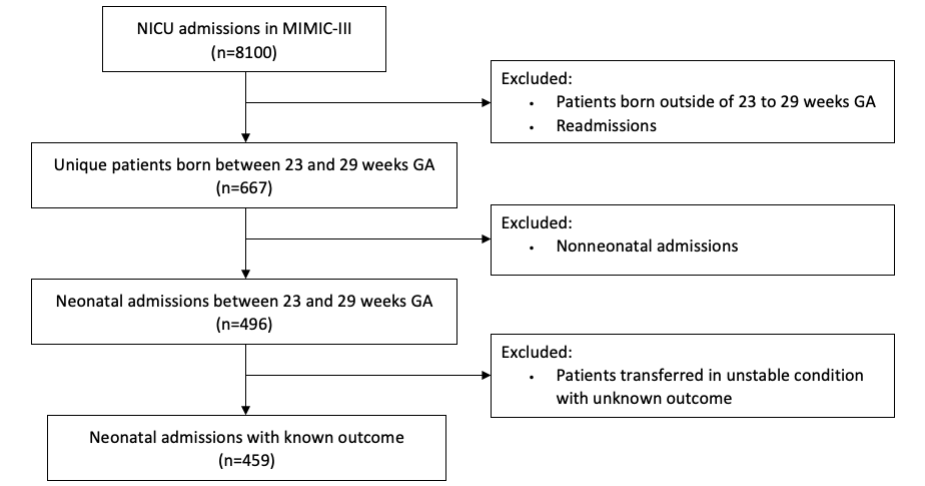
Flowchart of selection criteria. GA: gestational age; MIMIC-III: Medical Information Mart for Intensive Care III; NICU: neonatal intensive care unit.

Records of extremely preterm neonates admitted to the NICU in the MIMIC-III database were extracted using PostgreSQL (The PostgreSQL Global Development Group). A query was performed for admissions with *ICD-9* (*International Classification of Diseases, Ninth Revision*) codes corresponding to extremely preterm delivery less than 30 weeks as well as very low birth weight. From the resulting records, those of neonates born outside of 23 to 29 weeks were excluded, as well as duplicate records and readmissions. Some records corresponded to nonneonatal admissions, for example, where an infant had a prior history of preterm birth, and they were excluded. When the remaining records were reviewed, it was found that some neonates were transferred outside of the hospital for surgery and had an unknown outcome. These records were also excluded (Figure 1).

From the 459 neonatal admission records that were selected, the patients’ demographics, vital signs, laboratory results, medications, procedures, and clinical text were queried from the database and reviewed. Of the available information, relevant elements were extracted based on factors found to be pertinent in previous scoring systems and expert knowledge. By manually curating the clinical text, including completed admission and discharge notes, we were able to incorporate features found only in unstructured form, including maternal clinical comorbidities and pregnancy complications. For this study, consideration of neonatal assessment and treatment was limited to data found initially at the time of NICU admission. The nonnumerical elements were encoded. Data that varied by clinical severity were encoded in that order, and the remaining categorical data underwent binary encoding. Median imputation was used to complete missing data.

Ultimately, 83 features that could be used in machine learning algorithms were generated, of which approximately half represented maternal clinical and demographic information, with the remaining features representing infant findings at the time of admission (Multimedia Appendix 1).

### Model Analysis

Several machine learning classification algorithms were implemented using Python 3.8 scikit-learn 1.2, and the resulting models were tested for their efficacy in predicting mortality. The same algorithms were also examined considering only prepartum features, assuming birth weight would be an estimated weight, to produce models that could be of assistance for clinicians counseling patients prior to an extremely preterm birth.

The performance of each model was endeavored to be optimized. To ensure that feature value range did not drive performance, standard scaling as well as min-max scaling were applied to quantitative features and used for models that were dependent upon distance calculations (eg, logistic regression, neural network, and support vector machine [SVM]). The final reported models used standard scaling due to improved performance over min-max scaling. Scaling was not performed for models invariant to monotonic transformations, such as random forest [18]. For the decision tree–based models, the hyperparameters of number of trees and maximum depth were adjusted. Number of trees began at 50 estimators and was increased by 50 until performance plateaued, which was at 250 trees with a maximum depth of 6 for the random forest method and 350 trees with a maximum depth of 5 for AdaBoost. The k value in the k-nearest neighbor algorithm was adjusted from the default value of 3 up to 20 (approximating the square root of the number of samples), and performance peaked at 4 in the final model. Because of the expected relatively small and imbalanced class sizes (8.1% in the minority class), a held-out test set was not used, and 10-fold stratified cross-validation with an 80:20 training and testing ratio was performed to ensure similar ratios across folds [19]. Mean performance metrics for *F*_1_-score, area under the receiver operating characteristic (AUROC), and average precision are reported, as well as log loss and Brier score, where a smaller value is ideal when considering imbalanced classification.

Features most correlated with the predicted outcome were determined for the higher-performing methods. For the logistic regression model, coefficients most positively and negatively associated with mortality could be determined. For the remaining machine learning models, the most influential features were either directly queried using an available scikit-learn method or through the calculation of feature permutation importance.

## Results

Of the included neonatal patients, 37 of 459 (8.1%) expired during the admission period after birth. The average length of stay for infants who survived after initial admission was 62.5 (SD 37.3) days. The average gestational age of the neonates at birth was 27 (SD 1.67) weeks, and 236 (51.4%) were male versus 223 (48.6%) female. Birth weights ranged from 365 to 2165 g, with the average birth weight being 1016 (SD 278) g, and 441 neonates were considered to have a very low birth weight (<1500 g). The average maternal age was 31.4 (SD 6.02) years. In terms of race and ethnicity, the majority of the included infants were in a category considered to be White (n=278, 60.1%), followed by Black (n=69, 15%), unknown (n=42, 9.2%), other (n=25, 5.4%), Hispanic (n=25, 5.4%), Asian (n=16, 3.5%), and Native American (n=4, 0.9%; Table 1).

**Table 1:** Demographics of patients whose records were included in the study.

		Total (N=459), n (%)	Survived (n=422, 91.9%), n (%)	Expired (n=37, 8.1%), n (%)
**Gestational age (weeks)**				
	23	7 (1.5)	2 (28.6)	5 (71.4)
	24	40 (8.7)	28 (70)	12 (30)
	25	41 (8.9)	36 (87.8)	5 (12.2)
	26	52 (11.3)	49 (94.2)	3 (5.8)
	27	87 (19)	84 (96.6)	3 (3.4)
	28	106 (23.1)	98 (92.5)	8 (7.5)
	29	126 (27.5)	125 (99.2)	1 (0.8)
**Sex**				
	Male	236 (51.4)	214 (90.7)	22 (9.3)
	Female	223 (48.6)	208 (93.3)	15 (6.7)
**Race**				
	Asian	16 (3.5)	15 (93.7)	1 (6.3)
	Black	69 (15)	62 (89.9)	7 (10.1)
	Hispanic	25 (5.4)	23 (92)	2 (8)
	Native American	4 (0.9)	3 (75)	1 (25)
	White	278 (60.1)	255 (91.7)	23 (8.3)
	Other	25 (5.4)	23 (92)	2 (8)
	Unknown	42 (9.2)	41 (97.6)	1 (2.4)
**Insurance**				
	Private	343 (74.7)	311 (90.7)	32 (9.3)
	Government	116 (25.3)	113 (97.4)	3 (2.6)
	Uninsured	2 (0.4)	0 (0)	2 (100)
**Family religion**				
	Catholic	100 (21.8)	91 (91)	9 (9)
	Protestant	24 (5.2)	22 (91.7)	2 (8.3)
	Jewish	16 (3.5)	15 (93.7)	1 (6.3)
	Other	30 (6.5)	25 (83.3)	5 (16.7)
	Unknown	289 (63)	269 (93.1)	20 (6.9)
**Type of delivery**				
	Cesarean section	356 (77.6)	331 (93)	25 (7)
	Vaginal delivery	103 (22.4)	91 (88.3)	12 (11.7)
**Pregnancy type**				
	Singleton	247 (53.8)	230 (93.1)	17 (6.9)
	Multiple	212 (46.2)	192 (90.6)	20 (9.4)
**Antenatal steroids**				
	Received	369 (80.4)	347 (94)	22 (6)
	Partially received	71 (15.5)	65 (91.5)	6 (8.5)
	Not received	19 (4.1)	14 (73.7)	5 (26.3)

**Figure 2:**
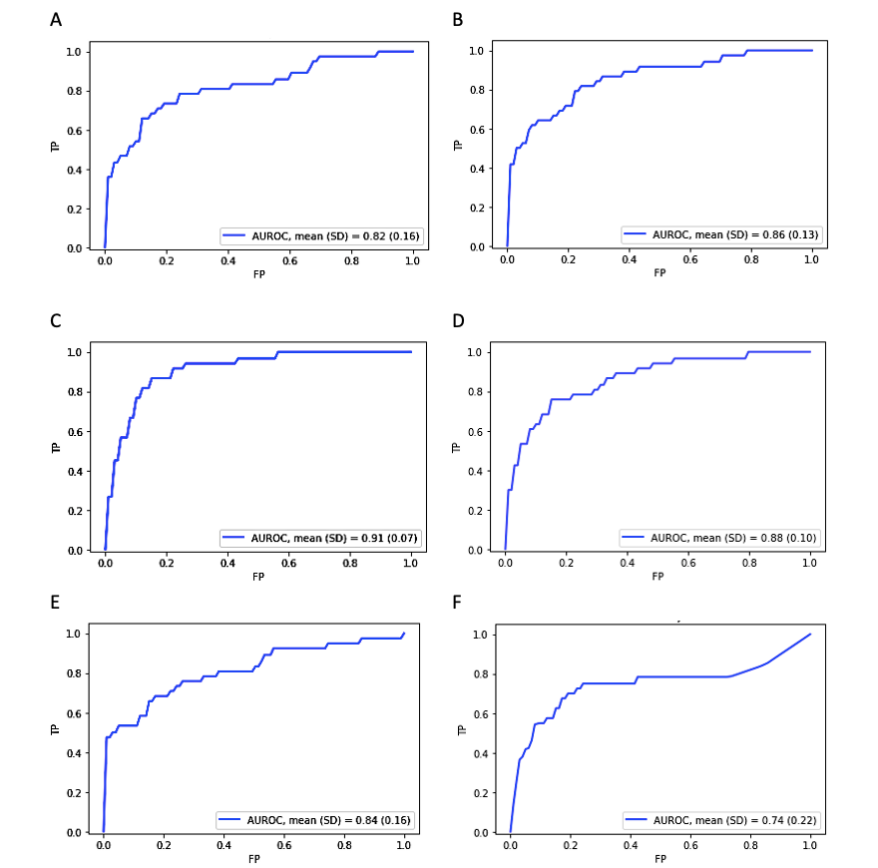
Receiver operating characteristic curves for the highest-performing models in Table 2. A: Logistic regression; B: SVM (support vector machine); C: Random forest; D: AdaBoost; E: Neural networks, F: Naïve Bayes; AUROC: area under the receiver operating characteristic; FP: false positive; TP: true positive.

Logistic regression, Naïve Bayes, k-nearest neighbor, SVM, random forest, AdaBoost, and neural network classifiers were compared for efficacy in predicting mortality (Figure 2 and Table 2). Standard scaling transformation improved performance only for the logistic regression, SVM, and neural network methods. The random forest model had the highest predictive performance when considering overall AUROC (mean 0.91, SD 0.07), *F*_1_-score (0.67), and Brier score (0.06). The AdaBoost model had the next highest AUROC (mean 0.88, SD 0.10); however, the *F*_1_-score (0.45) was low due to poor precision. On the other hand, the neural network model yielded the top *F*_1_-score (0.67) and Brier score (0.05) despite having a lower AUROC (mean 0.84, SD 0.16). SVM was overall next best performing model (mean 0.86, SD 0.13; *F*_1_-score 0.62; Brier score 0.06), followed by logistic regression (mean 0.82, SD 0.16; *F*_1_-score 0.61; Brier score 0.08). The Naïve Bayes (mean 0.74, SD 0.22; *F*_1_-score 0.40; Brier score 0.25) and k-nearest neighbor (mean 0.64, SD 0.13; *F*_1_-score 0.34; Brier score 0.07) methods were the worst performing.

**Table 2:** AUROC^a^, average precision, F1-score, log loss, and Brier scores for 10-fold stratified cross-validation predicting mortality using initial neonatal intensive care unit admission features (lower log loss and Brier scores are ideal when considering imbalanced classification).

Method	AUROC, mean (SD)	Precision, mean (SD)	*F*_1_-score	Log loss score	Brier score
Logistic regression	0.82 (0.16)	0.55 (0.25)	0.61	0.35	0.08
SVM^b^	0.86 (0.13)	0.61 (0.24)	0.62	0.20	0.06
Random forest	0.91 (0.07)	0.61 (0.22)	0.67	0.19	0.06
AdaBoost	0.88 (0.10)	0.55 (0.25)	0.45	0.80	0.07
Neural network	0.84 (0.16)	0.65 (0.24)	0.67	0.30	0.05
Naïve Bayes	0.74 (0.22)	0.39 (0.17)	0.40	3.90	0.25
K-nearest neighbor	0.64 (0.13)	0.24 (0.16)	0.34	1.74	0.07

^a^AUROC: area under the receiver operating characteristic.

^b^SVM: support vector machine.

On post hoc chi-square analysis of the categorical variables, the factors that most influenced the outcome were insurance status, initial breathing assessment of the infant, and presence of a serious fetal anomaly (Table 3). When examining Pearson correlation of continuous variables, higher levels of ventilation and blood pressure support as well as higher arterial blood gas base deficit were properties mildly to moderately correlated with mortality. Larger gestational age, birth weight, and higher APGAR (appearance, pulse, grimace, activity, and respiration) scores at birth negatively correlated with mortality to a similar degree (Table 4).

Similar features were most strongly associated with outcome in the machine learning–based models, although they varied in importance (Table 5). For example, in the random forest model, gestational age, birth weight, and initial oxygen level were of higher importance, whereas in the neural network model, initial blood pressure support and activity level were the most influential features.

Evaluation of classifiers using only prepartum features, assuming birth weight as the estimated weight, also yielded the highest performance measures with the random forest method (Table 6). The random forest features that were consistently of highest importance included gestational age, weight, and maternal age (Table 7).

**Table 3:** Chi-square: categorical features significantly associated with outcome.

Feature	Description	Chi-square (*df*)
un_ins	Uninsured	22.8 (1)
breathing1	Initial breathing assessment	21.4 (2)
anomaly	Serious fetal anomaly	20.8 (1)
airway1	Initial type of airway or ventilation	17.1 (4)
religion_jehovahs	Religion Jehovah’s Witness	11.4 (1)
twintwin	Twin-twin transfusion syndrome	9.4 (1)
uncertain	Uncertain pregnancy dating	7.9 (1)
religion_other	Religion other	4.9 (1)
gov_ins	Medicaid or Medicare insurance	4.7 (1)
muscle1	Muscle tone	4.6 (4)

**Table 4:** Pearson correlation: correlation of continuous features with mortality.

Feature	Description	Correlation
FiO2_1	Initial amount of oxygen ventilation	0.28
BD1	Initial arterial blood gas base deficit	0.23
dopa1	Initial IV^a^ dopamine rate	0.20
temp1	Initial temperature	0.14
pCO2_1	Initial arterial blood gas pCO_2_^b^	0.13
G	Maternal gravidity	0.07
P	Maternal parity	0.06
PRBC1	Initial IV blood transfusion amount	0.06
maternal_age	Maternal age	0.05
gluc1	Initial glucose	0.03
bands1	Initial bands	0.03
multiple	Number of fetuses at delivery	0.02
pO2_1	Initial arterial blood gas pO_2_^c^	–0.01^d^
wbc1	Initial white blood cells	–0.02
BPmean1	Initial mean blood pressure	–0.04
monos1	Initial monocytes	–0.05
HR1	Initial heart rate	–0.05
hct1	Initial hematocrit	–0.07
neuts1	Initial neutrophil count	–0.07
SaO2_1	Initial oxygen saturation	–0.20
birth_wt	Birth weight	–0.22
GA	Gestational age at birth	–0.32
apgar1	One-minute APGAR^e^ score	–0.32
apgar5	Five-minute APGAR score	–0.35

^a^IV: intravenous.

^b^pCO_2_: partial pressure of carbon dioxide

^c^pO_2_: partial pressure of oxygen.

^d^Negative correlations with mortality imply a correlation with survival.

^e^APGAR: appearance, pulse, grimace, activity, and respiration.

**Table 5:** Features of highest importance in various models, listed in order of importance. Positive and negative associations with mortality can be calculated only in logistic regression models. For the tree-based random forest and AdaBoost algorithms, an impurity-based method was used to determine overall feature importance. For the remaining algorithms, importance was found via feature permutation^a^.

Logistic regression: positively associated with mortality	Logistic regression: negatively associated with mortality	Random forest	AdaBoost	SVM^b^	Neural network
race_hispanic	GA	GA	neuts1	activity1	dopa1
color1	race_unk	birth_wt	hct1	GA	activity1
anomaly	apgar1	SaO2_1	SaO2_1	HTN	multiple
race_asian	gov_ins	BD1	wbc1	anomaly	uncertain
un_ins	activity1	apgar1	apgar1	breathL1	race_unk
dopa1	monos1	gluc1	monos1	breathR1	twintwin
abdomen1	breathL1	dopa1	temp1	twintwin	anomaly
pvt_ins	PRBC1	apgar5	HR1	birth_wt	muscle1
multiple	infert	FiO2_1	FiO2_1	antfont1	wbc1
FiO2_1	dm	neuts	bands1	caprefill1	abdomen1

^a^The descriptions of variable names are present in Multimedia Appendix 1.

^b^SVM: support vector machine.

**Table 6:** AUROC^a^, average precision, F1-score, log loss, and Brier scores for 10-fold stratified cross-validation predicting mortality when only prepartum features are available (lower log loss and Brier scores are ideal when considering imbalanced classification).

Method	AUROC, mean (SD)	Precision, mean (SD)	*F*_1_-score	Log loss score	Brier score
Logistic regression	0.77 (0.14)	0.41 (0.18)	0.51	0.29	0.07
SVM^b^	0.76 (0.10)	0.37 (0.15)	0.46	0.25	0.07
Random forest	0.80 (0.14)	0.54 (0.27)	0.59	0.22	0.06
AdaBoost	0.75 (0.17)	0.44 (0.29)	0.54	0.27	0.07
Neural network	0.76 (0.11)	0.44 (0.18)	0.53	0.31	0.07
Naïve Bayes	0.68 (0.21)	0.30 (0.11)	0.19	6.09	0.59
K-nearest neighbor	0.62 (0.12)	0.20 (0.12)	0.30	1.77	0.09

^a^AUROC: area under the receiver operating characteristic.

^b^SVM: support vector machine.

**Table 7:** Prepartum features of highest importance in various models, listed in order of importance^a^.

Logistic regression: positively associated with mortality	Logistic regression: negatively associated with mortality	Random forest	AdaBoost	SVM^b^	Neural network
maternal_age	GA	GA	birth_wt	GA	un_ins
anomaly	race_unk	birth_wt	maternal_age	steroids	steroids
un_ins	dm	maternal_age	GA	P	HTN
asthma	depression	anomaly	G	infert	GA
religion_jehovahs	PTL	G	multiple	G	anomaly
pvt_ins	steroids	P	religion_unk	uncertain	twintwin
race_hispanic	gov_ins	un_ins	steroids	birth_wt	race_unk
twintwin	HTN	steroids	sex	sex	sex
uncertain	infert	uncertain	anomaly	multiple	P
multiple	P	twintwin	P	anomaly	SVD

^a^The descriptions of variable names are present in Multimedia Appendix 1.

^b^SVM: support vector machine.

Several of the important features found in the top-performing models were among those manually curated in unstructured form, including the presence of maternal hypertensive disease and diabetes, uncertain pregnancy dating (uncertain), fetal anomaly (anomaly), and twin-twin transfusion syndrome.

## Discussion

### Principal Findings

There is a potential for existing risk calculators to be outperformed by tree-based machine learning algorithms, as indicated by the higher performance of our random forest model versus SNAPPE-II in the context of extremely premature or very low birth weight infants (in fact AUROC increased to mean 0.92, SD 0.05 when only the neonates <1500 g were considered in the random forest model to directly compare to SNAPPE-II). Performance difference compared with CRIB is inconclusive, however. In terms of estimating neonatal mortality prior to preterm birth, although the point estimates of several of the machine learning algorithms using additional features extracted from the EHR were higher than that of the NICHD calculator, overlapping CIs preclude any conclusion about significant differences in performance.

### Comparison to Prior Work

Examination of prior work further points to the importance of using data available from the EHR, including unstructured health data. For example, the relatively high-performing CRIB score includes the presence of fetal malformation as a variable. Saria et al [20] incorporated signal processing of short-term time series data from neonatal vital sign sensors to produce a model classifying infants at high risk for severe morbidity or mortality. To maintain accuracy over time, Meadow et al [11] proposed a longitudinal NICU survival model combining adverse events, imaging report information, and caretaker intuition. Hamilton et al [21] more recently applied tree-based machine learning in the context of preterm birth to determine clusters of pregnancy characteristics that were at the highest risk for severe neonatal morbidity or mortality.

### Strengths and Limitations

This study is limited by a small data set with data from a single institution, which in turn limits the ability to establish statistical significance in performance differences and the variety of machine learning methods that can be examined. Because of the retrospective nature of the study, there is less control over the format of the data and the amount of missing data. Although a single-institution data set is usually considered a limitation, Rysavy et al [4] emphasized that extremely preterm neonatal outcomes are significantly influenced by the hospital of birth and suggested maintaining ongoing and updated prediction models from outcomes within hospital systems. Using machine learning would be ideal for this task, allowing for consideration of a number of features retrievable from the EHR with a high tolerance for missing or outlier data as the volume of data increases. Tree-based machine learning algorithms may be additionally advantageous due to their ability to iteratively combine numerous weakly predictive features into stronger predictors.

Knowledge of the most influential features, which was possible to visualize in the majority of the presented models, provides transparency. Understanding which factors contribute most to the prediction of outcomes in a model can help clinical providers derive greater intuition regarding how applicable the model is to a particular patient.

The inclusion of maternal information and pregnancy characteristics found in unstructured form in the MIMIC-III database allowed for consideration of factors beyond the numerical neonatal data. Some of these additional variables, such as the presence of fetal anomalies or twin-twin transfusion syndrome, were found to be of high importance in several top-performing models, especially in those used in the prepartum period prior to an anticipated extremely preterm delivery. This illustrates that machine learning–based models could potentially be helpful for continuity of care, starting in the prepartum timeframe with ongoing predictive ability after birth. Maternal demographic information had an influence on mortality prediction in some of the higher-performing models but not others. Although demographic data can provide additional knowledge of social context, unintended bias can also be introduced into the resulting model [22].

### Future Directions

Future work anticipates further evaluation of these methods on larger, more diverse data sets to determine if there is a significant and reproducible performance advantage. Expanding the study to include additional data would also allow the evaluation of more powerful machine learning methods such as deep learning methods. Eventually, the maintenance of a more representative and up-to-date cohort for training could potentially be accomplished via collaborative or federated learning techniques across institutions [22,23]. To address the possibility of algorithmic bias, further work could include a comparison of prediction results using models with and without protected demographic features and a calculation of the level of discrimination that could result. Assessment of more data from underrepresented groups may also aid in producing increasingly accurate and less discriminatory models [24,25].

In this study, unstructured information was manually extracted from admission and discharge notes in the MIMIC-III database and allowed for consideration of additional relevant features in our models. This suggests that the use of natural language processing to better understand clinical context may further improve the prediction of outcomes of extremely preterm births. As automated natural language processing of clinical notes becomes more mature and prevalent, the use of these features gleaned from unstructured EHR data will be increasingly applicable [26].

Additional potential future directions include integrating with or adding functionalities found in other intensive care unit models, such as time series modeling, and predicting outcomes other than mortality, such as the development of comorbidities, discharge location, length of stay, and likelihood of readmission.

### Conclusions

This study examined machine learning models produced from the MIMIC-III NICU data set and their predictive ability in the clinically challenging situation of extremely preterm birth. The tree-based random forest model was found to have higher performance than the SNAPPE-II model when predicting the survival of extremely preterm infants of very low birth weight. Several other models, including those using only features that would be known prepartum, also appeared to have good predictive performance but failed to show a statistically significant difference from prior models. Features of highest importance in these models were explored and included traditional variables, such as gestational age and birth weight, but also information that may be found in unstructured form in the EHR. Evaluation of these and even more advanced machine learning methods on larger data sets may offer further clarity about performance differences, and natural language processing techniques would allow for greater use of unstructured clinical information.

## Appendix

Multimedia Appendix 1 Categorical and continuous features.

Multimedia Appendix 2 TRIPOD checklist for model development.

## Abbreviations

APGAR: appearance, pulse, grimace, activity, and respiration
AUROC: area under the receiver operating characteristic
CRIB: Clinical Risk Index for Babies
EHR: electronic health record
*ICD-9*: *International Classification of Diseases, Ninth Revision*
MIMIC-III: Medical Information Mart for Intensive Care III
NICHD: National Institute of Child Health and Human Development
NICU: neonatal intensive care unit
SNAPPE-II: Score for Neonatal Acute Physiology With Perinatal Extension II
SVM: support vector machine

## Data Availability

The data sets analyzed in this study are available in the Medical Information Mart for Intensive Care III (MIMIC-III) Clinical Database [14].
