# Supplementary material for: Improving Prediction of Survival for Extremely Premature Infants Born at 23 to 29 Weeks Gestational Age in the Neonatal Intensive Care Unit: Development and Evaluation of Machine Learning Models
Source: JMIR Med Inform. 2024 Feb 14;12:e42271. doi: 10.2196/42271 (PMC10902770; doi:10.2196/42271)
Supplement: Multimedia Appendix 1 [file medinform_v12i1e42271_app1.docx]

## Appendix

**Supplemental Table 1.** Categorical features

|  | Description | Values |
| --- | --- | --- |
| Demographics |  |  |
| delivery | Mode of delivery | CS (Cesarean Section), SVD (Spontaneous Vaginal Delivery), AVD (Vacuum or Forceps Assisted Vaginal Delivery) |
| insurance | Maternal insurance | pvt_ins (Private Insurance), gov_ins (Medicaid, Medicare), un_ins (Uninsured, Self Pay) |
| race | Maternal race | race_asian, race_black, race_hispanic, race_native, race_other, race_unk, race_white |
| religion | Maternal religion | religion_catholic, religion_christian_sci, religion_g_orthodox, religion_hindu, religion_jehovahs, religion_jewish, religion_other, religion_protestant, religion_r_orthodox, religion_unk |
| sex | Sex at birth | M, F |
| Neonatal |  |  |
| abdomen1 | Initial abdomen appearance | Distended, Full, Round, Scaphoid, Unknown, Flat |
| activity1 | Initial activity | Unassessable, Lethargic, Jittery, Unknown, Active |
| airway1 | Initial airway | Oral ET, Nasal ET, Nasopharyngeal, Nasal Prongs, Room Air |
| antfont1 | Initial anterior fontanelle | Bulging, Unknown, Flat |
| breathing1 | Initial breathing assessment | Grunt/Flare/Retraction, Unknown, Normal |
| breathL1 | Initial left sided breath sounds | Absent, Diminished, Coarse, Rales, Wheeze, Unknown, Clear |
| breathR1 | Initial right sided breath sounds | Diminished, Coarse, Rales, Wheeze, Unknown, Clear |
| BS1 | Initial bowel sounds | Not Audible, Hypoactive, Unknown, Active |
| caprefill1 | Initial capillary refill | Delayed, Unknown, Brisk |
| color1 | Initial color | Dusky, Cyanotic, Pale, Jaundice, Unknown, Mottled, Ruddy, Acrocyanotic, Pink |
| murmur1 | Initial heart murmur | Present, Unknown, Absent |
| muscle1 | Initial muscle tone | Unassessable, Hypotonic, Hypertonic, Unknown, Symmetrical |
| postfont1 | Initial posterior fontanelle | Bulging, Unknown, Flat |
| Maternal |  |  |
| PTL | Preterm labor | Yes, No |
| PPROM | Preterm premature rupture of membranes | Yes, No |
| HTN | Hypertensive disease | Yes, No |
| abrupt | Abruption, suspected abruption | Yes, No |
| asthma | Asthma | Yes, No |
| cerclage | Cerclage placement during pregnancy | Yes, No |
| cocaine | History of cocaine use during pregnancy | Yes, No |
| chorio | Chorioamnionitis | Yes, No |
| depression | History of depression on medication during pregnancy | Yes, No |
| dm | Diabetic disease | Yes, No |
| infert | Pregnancy following infertility treatment | Yes, No |
| smoker | History of smoking during pregnancy | Yes, No |
| steroids | Received antenatal steroids for fetal lung maturity | Yes, No |
| thyroid | Thyroid disease | Yes, No |
| uncertain | Uncertain dating due to poor or no prenatal care | Yes, No |
| maternal | Other maternal complication leading to preterm delivery | Yes, No |
| anomaly | Serious or life-threatening fetal anomaly | Yes, No |
| twintwin | Twin-twin transfusion syndrome | Yes, No |
| fetal | Other non-reassuring fetal status leading to preterm delivery | Yes, No |

**Supplemental Table 2.** Continuous features

|  | Description |
| --- | --- |
| Labwork |  |
| bands1 | Initial bands |
| BD1 | Initial arterial blood gas base deficit |
| gluc1 | Initial glucose level |
| hct1 | Initial hematocrit |
| monos1 | Initial monocytes |
| neuts1 | Initial neutrophils |
| pO2_1 | Initial arterial blood gas partial pressure of oxygen |
| pCO2_1 | Initial arterial blood gas partial pressure of carbon dioxide |
| wbc1 | Initial white blood cells |
| Treatment |  |
| dopa1 | Initial intravenous dopamine |
| FiO2_1 | Initial oxygen ventilation |
| PRBC1 | Initial transfusion |
| Monitoring |  |
| BPmean1 | Initial mean blood pressure |
| HR1 | Initial heart rate |
| RR1 | Initial respiratory rate |
| SaO2_1 | Initial oxygen saturation |
| temp1 | Initial temperature |
| Delivery |  |
| apgar1 | 1 minute APGAR score |
| apgar5 | 5 minute APGAR score |
| birth_wt | Birth weight |
| G | Gravida |
| GA | Gestational age |
| maternal_age | Maternal age |
| multiple | Number of fetuses at delivery |
| P | Para |
